# Correlated genetic effects on reproduction define a domestication syndrome in a forest tree

**DOI:** 10.1111/eva.12252

**Published:** 2015-03-21

**Authors:** Luis Santos-del-Blanco, Ricardo Alía, Santiago C González-Martínez, Luis Sampedro, Francisco Lario, José Climent

**Affiliations:** 1 Department of Forest Ecology and Genetics, INIA-CIFORMadrid, Spain; 2 Sustainable Forest Management Research InstitutePalencia, Spain; 3 Department of Ecology and Evolution, University of LausanneLausanne, Switzerland; 4 Misión Biológica de Galicia - CSICSalcedo, Spain; 5 Vivero de Maceda, Dirección Técnica, TRAGSAMaceda, Ourense, Spain

**Keywords:** adaptation, artificial selection, domestication syndrome, fitness traits, genetic change

## Abstract

Compared to natural selection, domestication implies a dramatic change in traits linked to fitness. A number of traits conferring fitness in the wild might be detrimental under domestication, and domesticated species typically differ from their ancestors in a set of traits known as the domestication syndrome. Specifically, trade-offs between growth and reproduction are well established across the tree of life. According to allocation theory, selection for growth rate is expected to indirectly alter life-history reproductive traits, diverting resources from reproduction to growth. Here we tested this hypothesis by examining the genetic change and correlated responses of reproductive traits as a result of selection for timber yield in the tree *Pinus pinaster*. Phenotypic selection was carried out in a natural population, and progenies from selected trees were compared with those of control trees in a common garden experiment. According to expectations, we detected a genetic change in important life-history traits due to selection. Specifically, threshold sizes for reproduction were much higher and reproductive investment relative to size significantly lower in the selected progenies just after a single artificial selection event. Our study helps to define the domestication syndrome in exploited forest trees and shows that changes affecting developmental pathways are relevant in domestication processes of long-lived plants.

## Introduction

Ever since Darwin, biologists have realized the opportunities brought about by domestication for the study of organic changes in all kind of organisms (Darwin 1868). Under domestication, individuals are diverted from natural selection processes into artificial selection conditions imposed by humans. Such conditions typically imply dramatic changes in the relationship between phenotypes and fitness (Meyer et al. 2012).

Darwin also coined the term ‘unconscious selection’, meaning the lack of intention of the breeder to modify the species. Nowadays, the meaning of the term also refers to correlated responses to selection on nontarget traits (Zohary 2004). Indeed, selected breeds do not only differ from their ancestors in only one target trait, but differences affect many correlated traits, all contributing to increase fitness under selection conditions, creating ‘domestication syndromes’ (Harlan 1971).

Therefore, during domestications, all traits providing fitness under natural conditions but not under domestication are likely to be selected against, either consciously or not. For instance, plant defence in natural environments is typically selected against during domestication (Meyer et al. 2012; Turcotte et al. 2014). This can be related to life-history evolution and the evolutionary concept of trade-offs, where maximum fitness is limited by negative correlations between traits (Roff 1992). As a result, depending on the degree of domestication, domesticated breeds will likely show reduced fitness or inability to survive outside domestication conditions. A similar coordinated response is observed as a result of management of wild populations by fisheries and fish farming (Hutchings and Fraser 2008) and perhaps also forest management (Sokol 2004). For instance, overexploitation of fisheries has caused strong genetic changes in exploited populations, driving the evolution of slow growing and reproductively precocious individuals (Olsen et al. 2004), revealing fundamental genetic correlations between growth rates and reproductive life-history traits.

Compared to other exploited organisms, studies on the domestication of forest trees for timber production are largely missing from the literature. This is perhaps because domestication of forest trees is still in its infancy compared to cultivated crops (Neale and Kremer 2011). Trees, and particularly monoecious conifers, provide a good model to study reproductive strategies and size-dependent sex allocation (Burd and Allen 1988). Therefore, the study of forest tree domestication from an evolutionary perspective can not only contribute to our understanding of plant reproductive ecology, but also shed light on the genetic basis for adaptation of these ecologically important species. Indeed, as key pieces determining structure and function of extensive terrestrial ecosystems (Petit and Hampe 2006; Whitham et al. 2006), even slight changes in the adaptive ability of individual trees can have broad consequences, but how and how much is currently unknown.

Common target traits in forest tree breeding are growth rate, timber yield, stem form and physical and chemical wood properties (Lepoittevin et al. 2011), all of them related to vegetative investment. Unintended correlated responses to selection are rarely reported in the forestry literature making it difficult to define a ‘domestication syndrome’ for forest trees (Cornelius 1994). Size at maturity and reproductive allocation are key fitness traits defining contrasting life histories in all kind of iteroparous long-lived organisms with indeterminate growth such as forest trees and exploited fishes (Roff 1992). As those traits imply shifting resource allocation between vegetative growth and reproduction, genetic breeding for allocation to growth is predicted to impact them.

Specifically, based on allocation theory, a shift in reproductive effort in the breeding population would be generally expected if the selection process is focused solely on growth traits (Roff 2000). Here we provide support for this hypothesis and evidence of this kind of correlated genetic effects during early domestication of a forest tree. We performed a long-term artificial selection experiment of *Pinus pinaster* (Maritime pine), a monoecious conifer widespread in south-western Europe in its early stages of domestication. Growth and reproductive traits of the progenies from phenotypically selected individuals were compared with those from control trees, not subjected to selection, growing together in a common garden in North Spain. More specifically, we tested the hypothesis that progenies from selected trees would reproduce at larger sizes and/or produce fewer cones at a given size. Besides, provided the greater cost in terms of growth for female reproduction, we hypothesized that female function of progenies selected for high stem growth rates should show a greater change, as compared to male function.

## Materials and methods

### Studied species and selection experiment

*Pinus pinaster* is a Western Mediterranean monoecious conifer, with a disjoint distribution in South-Western France, Iberian Peninsula (Portugal and Spain), Italy and North Africa (Morocco, Algeria and Tunisia). Significant within population additive genetic variation has been reported for growth, wood properties and stem form (Lepoittevin et al. 2011), resistance to herbivory (Zas et al. 2005; Moreira et al. 2012), and also for reproductive life-history traits (Santos-del-Blanco et al. 2010).

**Figure 1:**
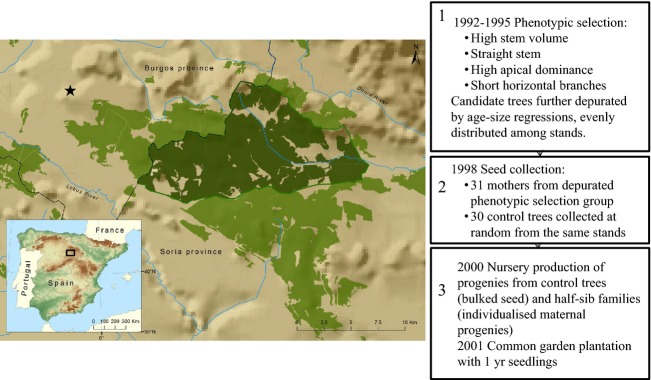
Location of the *Pinus pinaster* region of provenance Montaña de Soria-Burgos (light green), and the forest stand (dark green) where artificial selection for growth and timber yield was carried out. Boxes 1–3 describe the breeding programme from phenotypic selection to common garden establishment. Star denotes the location of the common garden.

A selection experiment was carried out in the ‘Montaña de Soria-Burgos’ provenance area, central-north Spain, during the 1990s. Thirty-one plus trees were selected in natural stands according to their superior phenotypes for growth and stem form, that is timber production. Altogether, <1 of 10 000 trees were selected as plus trees. Then, in 2001, their progeny was planted in a common garden together with the progeny from a control lot randomly selected within the same population. The common garden was placed in the vicinity of the surveyed stands (Latitude 41°55′15″N; Longitude 3°11′35″W; 1153 m a.s.l.; Fig.1). The design comprised 28 complete blocks and single-tree plots. A detailed description of the selection protocol and common garden design can be found in the Appendix S1.

A total of 1087 trees were included in this study, 833 belonging to progenies of selected trees (selected group) and 254 to progenies of unselected trees (control group). Final sample size was 868 trees due to mortality (2%) and biotic damage (18%). We confirmed both the absence of any bias due to these sample reduction and any additional effect of differential competence not accounted for in further analyses.

### Field measurements and variable description

Two measurements were carried out in late May in 2009 and 2011, when the trees were eight and ten years old, respectively. In both dates, we measured tree height below the elongating bud (this is, previous winter height) and diameter at breast height (for those trees ≥1.3 m) and female cones were counted for the following cohorts: female strobili (red-coloured and placed at the end of the current year flush), one-year-old immature conelets and two-year-old and above mature cones (Fig. S2). Male (pollen) cone abundance was categorically scored from 0 (absent) to 3 (very abundant). Stem form and branching habit was assessed through an ordinal scoring from 1 to 6 in 2009 (Raymond and Cotterill 1990). A higher score implied high apical dominance and straight stem with few branches, inserted at high angles (closer to horizontal position) (Galera et al. 1997). Total biomass was estimated from diameter at breast height using standard formulae in Montero et al. (2005).

Considering its reproductive status, each tree was classified as either reproductive or nonreproductive. According to their first reproductive event, trees were assigned to female (F, those that started their reproductive life as females, i.e. ontogenetically protogynous), male (M, those that started their reproductive life as males, i.e. ontogenetically protandrous) or cosexual (C, those that started their reproductive life with both female and male strobili) groups. Nonreproductive trees were further classified as juveniles (J, size below the family or group-specific smallest reproductive individual) or vegetative (V, size above the family or group-specific smallest reproductive individual).

### Statistical analyses

We used Bayesian approaches for fitting quantitative genetic models (Nakagawa and Schielzeth 2010). We computed posterior mode and 95% credible intervals (95% CIs) for fixed effects, variance components, threshold sizes for reproduction, heritabilities and phenotypic correlations across a variety of models. A detailed description of priors used can be accessed in the Appendix S2. Analyses were run in R, version 2.15.2 (R Development Core Team, Vienna), using the package MCMCglmm, version 2.01 (Hadfield 2010).

Tree height at ten years was modelled as a normal distributed trait with Gaussian errors including block as a random factor. Stem form was modelled as an ordinal trait with a generalized model with probit link and residual variance (*V*_*R*_) fixed to one by convention (e.g. Reid et al. 2011). Stem form model included block as a random factor. Fixing *V*_*R*_ to a particular value affects proportionally all variance components in a model, and thus, heritability estimates are independent of the *V*_*R*_ value.

Median threshold size for first reproduction (TSFR) was calculated as the result of dividing intercept by slope estimates from a generalized binomial model with logit link, where categorical reproduction (present or absent) at eight and ten years was the response variable. As slope estimates are always negative because the probability of reproduction increases with size, they were included as absolute values to generate positive TSFR estimates (Wesselingh and Klinkhamer 1996; Mendez and Karlsson 2004). Height was included as a covariate (Wesselingh and de Jong 1995). *V*_*R*_ was fixed to one in binomial models by convention. Cumulative quantitative female reproduction, that is number of cones produced throughout a tree's life, was modelled as a Poisson generalized model with log link, using log biomass at ten years as a continuous predictor (Female R-V). This was equivalent to standard log–log allometric Reproductive versus Vegetative size (R-V) regressions but benefited from the inclusion of zeroes in the response variable (nonreproductive individuals), otherwise commonly discarded or transformed (Kotze and O'Hara 2010). Only data from nonjuvenile trees were used in R-V models to avoid zero inflation (Mendez and Karlsson 2004). Male reproduction at ten years, an ordinal trait, was modelled analogously to stem form, but including tree log biomass as a covariate (Male R-V). Inclusion of size (height or biomass) as a covariate in TSFR and R-V models accounts for small scale environmental variation in common gardens (Santos-del-Blanco et al. 2012), and so, block effects were not considered.

### Direct and indirect effects of early domestication

We first tested for differences between progenies from the selected (hereafter ‘selected group’) and nonselected (hereafter ‘control group’) mother trees. We analysed height, stem form, threshold for first reproduction (TSFR), and reproductive–vegetative (R-V) size relationships for female and male reproduction by fitting independent univariate models and using fixed effects (selected–control) 95% credible intervals (95% CIs) to evaluate the significance of their difference. Model specification can be found in the Appendix S3. Average sizes per reproductive group (juvenile, vegetative, female, male or cosexual) were also calculated. For TSFR and R-V models, we evaluated the significance of separate additive (intercept) and multiplicative (slope) selection effects.

### Genetic control and correlation among traits

To evaluate the quantitative genetic basis of variation in traits affected by early domestication, we fitted ‘animal models’ to data from the selected group to estimate additive genetic variance. A detailed description of model specification and estimation of quantitative genetic parameters can be accessed in the Appendix S3.

As 95% CIs for variance components cannot overlap 0, it is not possible to test the null hypothesis of zero variance. Instead, significance of variance components was assessed by means of deviance information criteria (DIC), comparing DIC values of nested models (Spiegelhalter et al. 2002). However, this was carried out only for linear mixed models (height) as interpretations of DIC in generalized linear mixed models with latent variables may not be clear. In these cases (binomial, Poisson and ordinal models), parameter posterior distribution was used to illustrate the magnitude of variance components (see Reid et al. 2011).

Genetic correlations were estimated as the Pearson correlation between traits for family corrected means derived from mixed models where family was coded as random (Lamy et al. 2012). Phenotypic correlations were estimated by fitting bivariate models to data from the selected and control groups.

## Results

### Direct and indirect effects of domestication

**Figure 2:**
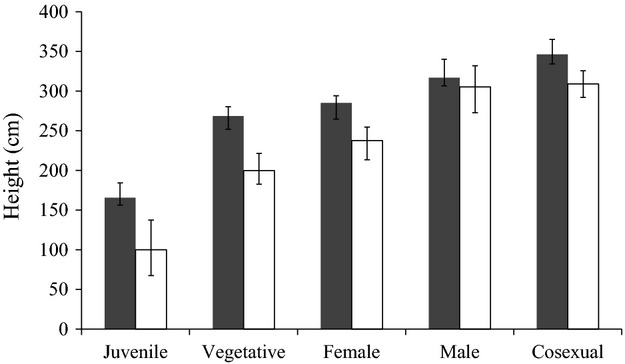
Effect of a single event of artificial selection for growth and timber yield on the height of the progeny from selected *Pinus pinaster* trees compared to an unselected control group. Bars represent average height at age 10 years for selected progenies (dark bars: *n* = 656) and the control, unselected group, representing the base population mean (white bars; *n* = 195). Results are presented for protogynous, protandrous and cosexual groups, as well as for vegetative–juvenile groups. Error bars represent 95% credible intervals. ‘Vegetative’ refers to those nonreproductive trees taller than the smallest reproductive tree for a given group.

Progenies of trees selected for timber yield (hereafter ‘selected group’) were significantly higher at age 10 than those sampled at random from the wild base population (hereafter ‘control group’; average height difference between selected and control trees: 11.7 cm) (Table1). As expected, phenotypic selection for timber yield leads also to better stem form scores (more straight stems) in the selected than in the control group (*P* < 0.001; Table1). Trees from the selected group were consistently taller than those in the control group for juvenile, vegetative, female and cosexual groups (all *P* < 0.05; Fig.2). However, height of early male trees did not significantly differ between groups and showed large variability within groups (Fig.2, Table S1).

**Table 1:** Effects of a single artificial selection event aimed at improving growth and timber yield in a *maritime pine* wild population. Selected and unselected groups were grown in a common garden close to the original population in central-north Spain. Effect size and 95% credible interval, CI, are shown. For models where a covariate was used (height or biomass), we indicate both additive (add., intercept) and multiplicative (mult., slope) effects of selection. Results are reported on the corresponding latent linear scale

Trait		Effect size	95% CI		*P*-value
Height		11.7	1.9	27.7	**0.024**
Stem form		0.375	0.218	0.618	**<0.001**
Female TSFR	add.	−1.242	−1.452	−0.893	**<0.001**
	mult.	−0.003	−0.006	0.001	0.198
Female R-V	add.	−1.627	−1.918	−1.197	**<0.001**
	mult.	0.123	−0.128	0.301	0.45
Male TSFR	add.	−1.616	−1.957	−1.272	**<0.001**
	mult.	−0.002	−0.007	0.003	0.422
Male R-V	add.	−0.886	−1.278	−0.605	**<0.001**
	mult.	−0.073	−0.274	0.176	0.764

TSFR, threshold size for first reproduction; R-V, relative reproductive–vegetative effort obtained from a Poisson (female) or ordinal (male) model with number of cones (female) or qualitative pollen production (males) as the response variable and log (biomass) as a covariate.

Bold values indicate significant values.

**Figure 3:**
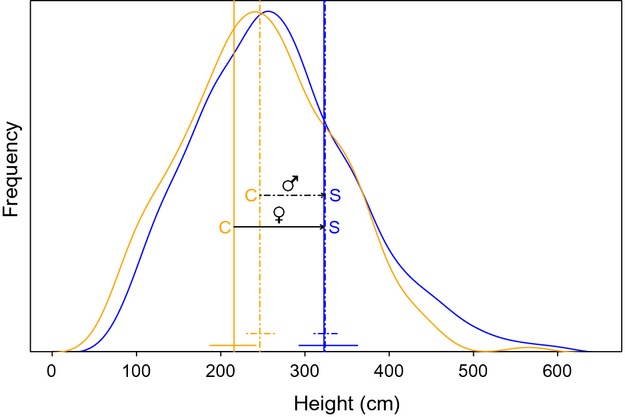
Comparison of height density distribution and threshold size (height) for first reproduction between *Pinus pinaster* progenies of trees either selected (S) or not selected (C, control) for timber production after one generation. Bell-shaped lines represent height probability distribution at age 10 years. Control group, orange lines; selected group, blue lines. Vertical lines represent threshold sizes for reproduction. Solid line, female function; dashed line, male function. 95% credible intervals for threshold sizes are represented by horizontal lines. Arrows show changes in threshold size for reproduction in male and female function due to selection. Control group, left; selected group, right.

The most prominent differences between the selected and control group were observed in reproductive traits, leading to delayed reproduction both in age and size terms in the former (Table1). The proportion of reproductive trees was higher in the control group (66%) than in the selected one (51%) (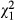 14.3 *P* < 0.001). In both groups, trees started their reproductive phase most commonly as females, followed by cosexuals and then males (Table S1). Among reproductive individuals, the selected group had a lower proportion of cosexuals (36%) (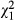 13.2 *P* < 0.001) and higher proportion of males (24%) (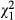 14.3 *P* < 0.007) compared to the control group (54% and 13%, respectively) (Table S1). Differences in reproductive traits between both selected and control groups were highly significant (*P* < 0.001, Table1). The selected group had significantly greater female and male threshold size for first reproduction (TSFR) and showed a consistently lower allocation to reproduction at a given size (reproductive–vegetative allocation, female and male R-V; Table1). The maximum change in TSFR was recorded for the female function, which increased in 106.9 cm in height in the selected population (Fig.3). In the control group, female TSFR was significantly lower than male TSFR (Fig.3, Table S1), but both parameters did not differ in the selected group as selection had a greater effect on female TSFR (Fig.3, Table S1). The control group showed the smallest reproductive individual and significantly lower male TSFR than any of the 31 selected progenies. Regarding the female TSFR, all but two selected families showed point estimates above the control, but wider credible intervals made those differences nonsignificant for about half of the selected families (Fig. S1).

### Quantitative genetic parameters of growth and reproductive traits

Among the selected progenies, additive genetic variance for height was very low compared to total variance, with a heritability of 0.06 (CI 0.02–0.17) (Table2). Heritability for stem form was 0.13, significantly different from zero. Narrow sense heritabilities were sharply different between female and male reproduction. While those of female TSFR and relative reproductive investment (R-V) were very high (>0.53), those for male function were indistinguishable from zero (Table2).

**Table 2:** Posterior modes (*h*^*2*^) and credible intervals (95% CI) of narrow sense heritabilities and variance components (*V*_*A*_ additive genetic variance, *V*_*I*_ additive overdispersion variance, *V*_*R*_ residual variance, *V*_*L*_ latent scale variance) for growth and reproductive traits recorded on a population of Maritime pine. Values for stem form, TSFR and R-V are reported on the corresponding latent linear scale

Trait	*h* ^2^	95% CI		*V*_*A*_	95% CI		*V*_*I*_	95% CI		*V*_*R*_	95% CI		*V*_*L*_
Height	0.06	0.02	0.17	372	121	1188				6376	5656	7513	
Stem form	0.13	0.03	0.36	4.7	0.8	13.3	22.2	11.4	36.9	1			1
Female TSFR	0.53	0.35	0.91	23.0	9.6	38.7	11.0	0.0	22.3	1			*π*^2^/3
Female R-V	0.73	0.42	0.81	2.1	1.0	2.9				0.2	0.0	0.9	
Male TSFR	0.00	0.00	0.24	0.02	0.00	4.38	10.24	4.43	18.2	1			*π*^2^/3
Male R-V	0.00	0.00	0.43	0.01	0.00	1.52				1			1

TSFR, threshold size (height) for first reproduction; R-V, relative reproductive–vegetative effort obtained from a Poisson (female) or ordinal (male) model with number of cones (female) or qualitative pollen production (males) as the response variable and vegetative size as a covariate.

In the selected group, we found significant negative genetic correlations between female TSFR and female relative reproductive investment (*r* = −0.82; Table S2a) indicating that precocious trees were also more prolific at this stage. Male and female thresholds for first reproduction were positively correlated (*r* = 0.39) but not the relative investment in male and female functions (Table S2a). Within the 31 selected families, we only found evidence of genetic trade-offs between height and reproduction for male function, but not for female function (Table S2a).

## Discussion

In our study, we found that phenotypic selection pursuing an ideotype of tall, thick straight trees with short horizontal branches and high apical dominance caused a sharp ontogenetic delay, even when reproductive investment was disregarded as a selection criterion.

There was a positive phenotypic correlation between growth and absolute female and male reproduction (Table S2). However, due to ‘unconscious selection’ (Darwin 1859) of traits genetically correlated with increased stem allocation, female and male threshold sizes for reproduction were both increased and male and female reproductive allocation decreased in the selected group. The phenotypic change due to this single selection event was as high as half of the species range of the threshold size for female reproduction and similar to the species range of the threshold size for male reproduction (1.0 m female, 0.8 m male) (Santos-del-Blanco et al. 2012) (Fig. S1). This shift revealed a likely underlying negative genetic correlation between allocation to growth and reproduction in the base population (Schluter 1996). Differences in ontogenetic development were also reflected in a higher proportion of reproductive individuals in the control group compared to the selected group. Assuming current growth rates, this implied a delay of between two and three years in the reproductive ontogeny of the selected group, compared to the control group. Thus indirect selection effects on male and female reproductive traits caused delayed reproduction both in age and size terms. Altogether, direct and indirect effects of selection contributed to define a ‘domestication syndrome’ for timber production in conifer trees.

In general, female reproduction in plants has been usually found to be more costly than male reproduction (Obeso 2002). In agreement with this idea, we found smaller average size for ontogenetically protogynous than protandrous trees in maritime pine, indicative of higher female reproductive costs (Fig.2). Despite the fact that we only detected negative genetic correlations between male reproduction and growth, our results indicate that strong negative genetic association between growth and early male and female reproductive effort existed in the native original population subjected to selection. At the genetic level, greater costs of female reproduction could be thought of as stronger negative correlations for growth (Reznick 1985), thus, a more intense indirect change of the threshold size for female reproduction, compared to the male threshold, fitted our expectations.

Other studies have reported on a related issue, namely the genetic consequences of selective harvesting in natural populations. The most paradigmatic examples come from fisheries (Koskinen et al. 2002), but a few examples also focus on plant species, mostly tree species. Here, as it happens with fish species, nonharvested individuals tend to show lower growth rates as in *Picea rubens* (Sokol 2004) or *Panax quinquefolius* (Mooney and McGraw 2009), but no reproductive data were reported. On the other hand, poor growth of *Eucalyptus* landraces in India has also been linked to selection for increased seed production (Varghese et al. 2009), where local people collect seeds for trading and thus likely select on increased fertility. Overall, this limited evidence together with our results are consistent with a investment in growth versus investment in reproduction trade-off, reflected both ways, that is selecting for increased growth indirectly selects for reduced fecundity, and selecting for increased fecundity indirectly selects for reduced growth.

In nature, reproductive traits and fitness are closely related (Stearns 1992). Even when an accurate measure of fitness in forest trees is challenging, reproductive traits have shown signs of local adaptation in *P. pinaster* (Santos-del-Blanco et al. 2012). It is doubtful whether in nature, control individuals from our experiment could have greater or equal fitness than selected progenies, but clearly a single severe perturbation (like a wildfire) would be more detrimental for the recruitment of domesticated, selected genotypes due to their immaturity (Keeley et al. 1999). If our results also hold true for other forest tree species, this reinforces the need for conservation of standing genetic diversity. This would ensure the evolutionary potential facing intensive breeding of native forest species (Koskela et al. 2007), particularly considering the challenges and uncertainties from climate change.

Lastly, in addition to being a valuable tool in reproductive ecology, selection experiments like this can also be a powerful tool to unveil the genetic basis of adaptation to both natural and human environments (Ross-Ibarra et al. 2007), highlighting phenotypic, quantitative genetic and genomic differences between domesticated and wild populations (Harfouche et al. 2012). Our study shows that changes affecting developmental pathways hold great interest for a better understanding of domestication.

## Data accessibility

Data are accessible through the following URL: http://hdl.handle.net/10532/2800 and http://www.genfored.es/app/metadata/data_details.php?id=32.

## Supporting Information

**Appendix S1.** Phenotypic selection in the wild and sampling. **Appendix S2.** Prior specification for statistical models. **Appendix S3.** Model specification and estimation of quantitative genetic parameters. **Figure S1.** Effect of a single event of artificial selection for timber yield within a *Pinus pinaster* population on the threshold size for first reproduction of the progeny of selected plus trees and control, unselected, base population. **Figure S2.** Description of *Pinus pinaster* seed cone development. **Table S1.** Posterior mode values and credible intervals (95% CI) of height (cm) per reproductive class (juvenile, vegetative, female, male or cosexual) and experimental group (selected, control) in *Pinus pinaster* trees. **Table S2.** Genetic and phenotypic correlations between vegetative and reproductive traits measured in *Pinus pinaster* trees grown in a common garden in North Spain.
