# Supplementary material for: Correlated genetic effects on reproduction define a domestication syndrome in a forest tree
Source: Evol Appl. 2015 Mar 21;8(4):403–10. doi: 10.1111/eva.12252 (PMC4408150; doi:10.1111/eva.12252)
Supplement: Supplementary file 1 [file eva0008-0403-sd1.docx]

**SUPPLEMENTARY TEXT 1. Phenotypic selection in the wild and sampling**

A breeding program for the *Pinus pinaster* region of provenance Montaña de Soria-Burgos in Northern Spain (Alía *et al.* 1996) was designed in the 1990’s. The first stage involved phenotypic selection of plus trees growing in a natural stand. Best valued traits were high stem volume growth, straight stem, high apical dominance and short branches inserted at high angles (closer to horizontal position) (White *et al.* 2007). Trees were selected between 1992 and 1995 by subjective comparison with neighbouring individuals. In order to avoid biasing the selection of best phenotypes towards favourable sites, a geographical subdivision of the stand was made, such that best scoring individuals of all subdivisions were chosen for progeny testing. All individuals were above 28 years old (at breast height), between 20 and 28 m tall and between 35 and 60 cm of diameter over bark at breast height. Some candidate plus trees had to be discarded as they did not produce enough seed for progeny testing. Considering the area of sampled stands and the average tree density, less than 1 out of 10,000 trees were selected as plus trees. The history of forest management in the area and the age of all mother trees allowed us to discard seed transfers from unknown origins.

Ripe cones of 31 selected plus trees were collected in autumn 1998. Also, a control seed lot was built the same year from cones collected randomly from 30 trees felled during forest management activities. This ensured that control seed lot was representative of the average phenotype of the study stand, avoiding a frequent bias of commercial seed lots collected from standing trees, therefore over-representing highly reproductive mothers (Varghese *et al.* 2009). The control seed lot was built in the same stand where plus tress were selected. This ensured that comparisons were not biased by fine-scale local adaptation and that pooled data from these 31 plus tree progenies gathered a priori a similar genetic variability with that of the control seed lot.

All cones harvested from selected and control trees were processed together. Cones were opened by exposing them to 60º C during one hour in an oven after air-drying for a week. Seeds were extracted manually, and kept dry at 4ºC until sowing in 2000. Seedlings were produced in 250 cc containers with a mixture of fertilized peat and vermiculite following best nursery practices for the species and planted in the common garden in 2001. Plants were placed at the intersections of a 3 x 3 m grid following a resoluble alpha design with 28 complete blocks and single-tree plots.

The common garden for progeny testing was set in a former marginal arable land, within the area where phenotypic selection was carried out (Figure 1). The plot is placed at 1.153 m above sea level, with approximately 10% of slope facing eastwards. The mother rock is siliceous sandstone and conglomerates, the predominant soil type in the ‘Montaña de Soria-Burgos’ area, but soil impoverishment and erosion from inadequate previous farming practices were evident.

**SUPPLEMENTARY TEXT 2. Prior specification for statistical models**

Default priors for fixed effects followed a normal distribution centred around cero and had a large (10^8^) variance. Non-informative diffuse proper priors were used for variance components. By default, we used inverse-Wishart priors with parameter V = 1 and a degree of belief of 0.002. However, given the null or very low value of some variance components, parameter expanded priors were also used in order to improve chain mixing. For height mixed model, the working parameter prior was normally distributed, with a mean of 0 and a variance of 1000, and the location effect prior was inverse-Wishart distributed, forming a scaled non-central F distribution, with a degree of belief parameter and a limit variance of one. In the case of binomial (male and female threshold size for reproduction) and ordinal models (stem form and male reproduction) residual variance was fixed to 1, causing inflation in heritability estimates due to high additive genetic variances driven by long tails in prior distribution. In order to keep additive genetic variance within expected boundaries, we used a Chi-square distributed prior with one degree of freedom for variance components (de Villemereuil *et al.* 2013). All MCMC analyses were run yielding in every case a total of 1,000 data points for each analysis and ensuring autocorrelations below 0.1.

**SUPPLEMENTARY TEXT 3. Model specification and estimation of quantitative genetic parameters**

Model specification for the estimation of a) direct and indirect selection effects and b) quantitative genetic parameters.

| **Trait** | **Link function** | **Fixed** | **Random** |
| --- | --- | --- | --- |
| a) |  |  |  |
| Height or biomass | Gaussian | Treatment | Block |
| Stem form | Probit | Treatment | Block + individual |
| Female TSFR | Logit | Treatment x height | Individual |
| Female R-V | Poisson | Treatment x biomass | None |
| Male TSFR | Logit | Treatment x height | Individual |
| Male R-V | Probit | Treatment x biomass | None |
| b) |  |  |  |
| Height or biomass | Gaussian | None | Animal + block |
| Stem form | Probit | None | Animal + block + individual |
| Female TSFR | Logit | Height | Animal + individual |
| Female R-V | Poisson | Biomass | Animal |
| Male TSFR | Logit | Height | Animal + individual |
| Male R-V | Probit | Biomass | Animal |

Animal models use pairwise coefficients of relatedness (0.25 between half-sibs) among individuals to define a matrix that is proportional to the variance-covariance structure of additive genetic effects. In these models, individual (animal) effects estimate the variance in phenotypes that can be related to additive genetic variance (V_A_). For stem form and TSFR generalised models, an additional individual effect was added to account for overdisperssion, i.e. individual variance (V_I_) larger than that specified by the model link due to causes other than additive genetic effects.

The heritability of normally distributed growth variables (height and biomass) was calculated by dividing V_A_ by total variance (V_A_ + residual variance, V_R_). Latent scale heritability values can be interpreted as heritability estimates of continuous, directly immeasurable variables that describe variation in discontinuous traits. For example, increasing continuous values of liability for reproduction are translated into two phenotypes (reproduction absent or present) separated by a threshold. Heritability of tree form and male reproduction on the underlying (probit) latent scale, was calculated as: h^2^ _form latent_: V_A_ / (V_A_+V_I_+V_R_+V_L_), and h^2^ _RVm latent_: V_A_ / (V_A_+V_R_+V_L_), respectively, with V_R_ = 1 by convention and latent scale variance V_L_ = 1 as the probit variance. Heritability of the threshold size for female and male reproduction was also calculated on the underlying (logit) latent scale as: h^2^ _TSFR latent_: V_A_ / (V_A_+V_I_+V_R_+V_L_), with V_R_ = 1 by convention and V_L_ = π2/3 as the logit variance. Heritability on the underlying (Poisson) latent scale for female reproductive investment after accounting for size was calculated as:

h^2^ _RVf latent_: V_A_ / {V_A_+V_R_+log[1/exp(β_0_)+1]}, where β_0_ is the predicted trait value given certain fixed effects (Nakagawa & Schielzeth 2010). We calculated h^2^ _RVf latent_ taking β_0_ as the average cone production per tree.

Genetic correlations were estimated as the Pearson’s correlation between traits for family means derived from mixed models where family was coded as random (Sakai *et al.* 2008; Lamy *et al.* 2012). Genetic correlations were only calculated for those variables with significant genetic variance or heritability. Phenotypic correlations were estimated by fitting bivariate models to data from the selected and control groups. Models were fitted with two variables at each time given that there were slight differences in number of observations per variable due to the existence of juveniles regarding male or female function. The variables included were height, biomass, stem form and male (qualitative scale) and female (cone counts) reproduction. Height and biomass were considered Gaussian variables while quantitative female reproduction was treated as Poisson. Originally, stem form and male reproduction were recorded according to an ordinal distribution, but here were considered also as count data (Poisson error) in order to enable the calculation of phenotypic correlations (Hadfield 2010). Genetic and phenotypic correlations thus do not represent the same set of traits as phenotypic correlations for male and female reproductive data were not corrected for size effects.

**SUPPLEMENTARY FIGURE 1.** Effect of a single event of artificial selection for timber yield within a *Pinus pinaster* population on the threshold size for first reproduction of the progeny of selected plus trees and control, unselected, base population. Smallest reproductive individual (black dots), and threshold size for first female (grey squares) and male reproduction (empty squares) per family and control group are shown. Family or group mean ± 95% credible intervals are shown. Control, control group; 26P-52S codes, half-sib family codes; SW Female and Male: female and male Species-Wide ranges for the threshold size for first reproduction (reported by Santos-del-Blanco L, Climent J, González-Martínez SC, Pannell JR (2012) Genetic differentiation for size at first reproduction through male versus female functions in the widespread Mediterranean tree *Pinus pinaster*. Ann Bot 110:1449–1460)

**SUPPLEMENTARY FIGURE 2.** Description of *Pinus pinaster* seed cone development. Left, current year female strobilus. Mid-left, first year female cone. Mid-right, second year mature female cone. Right, whorls of serotinous female cones older than two years attached to the main stem.


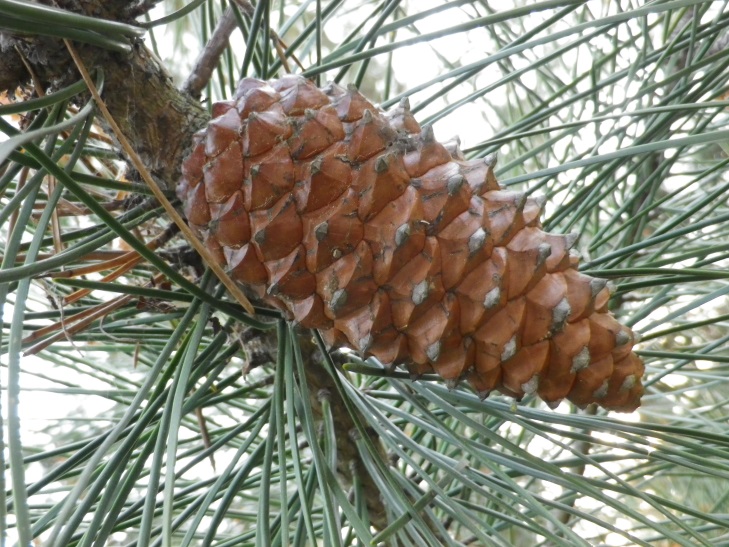

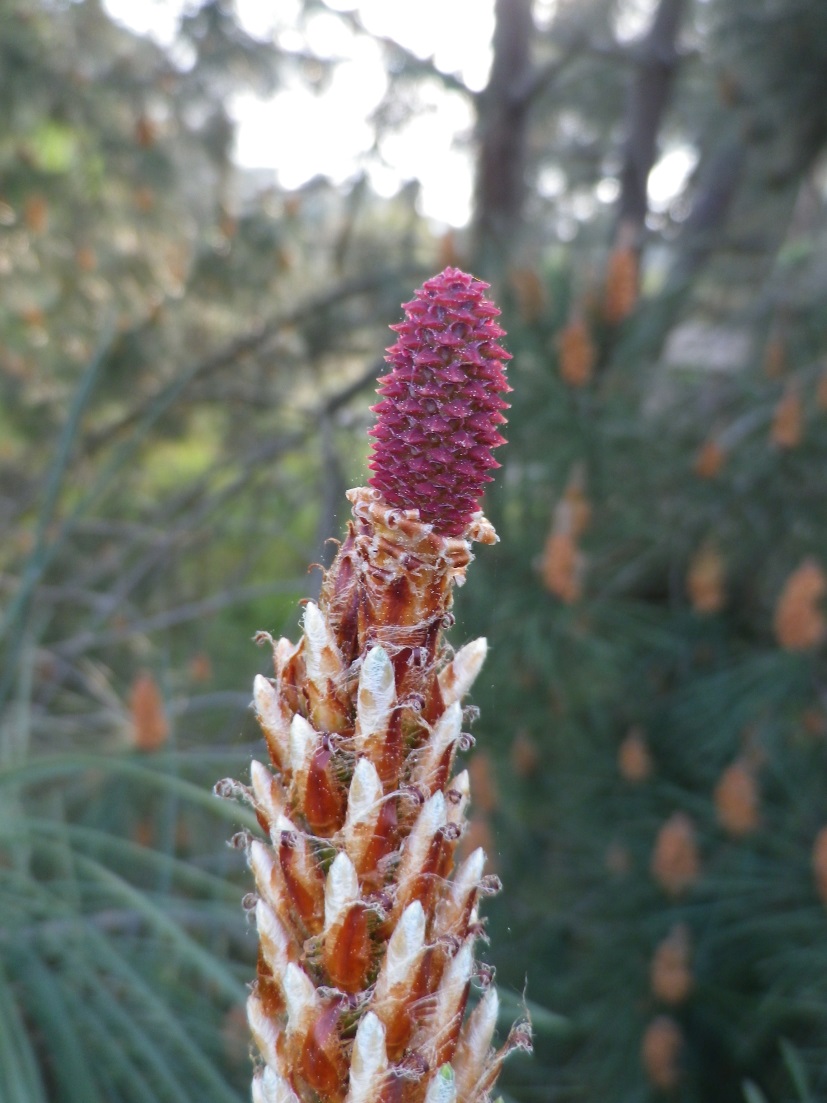

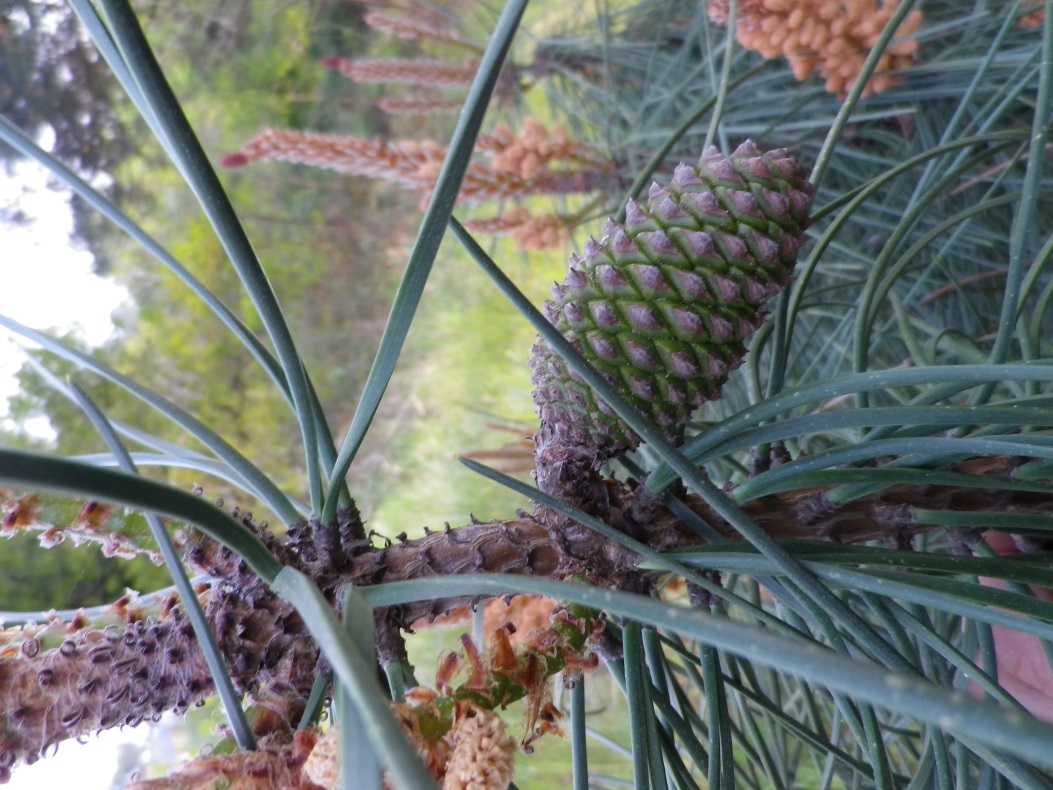

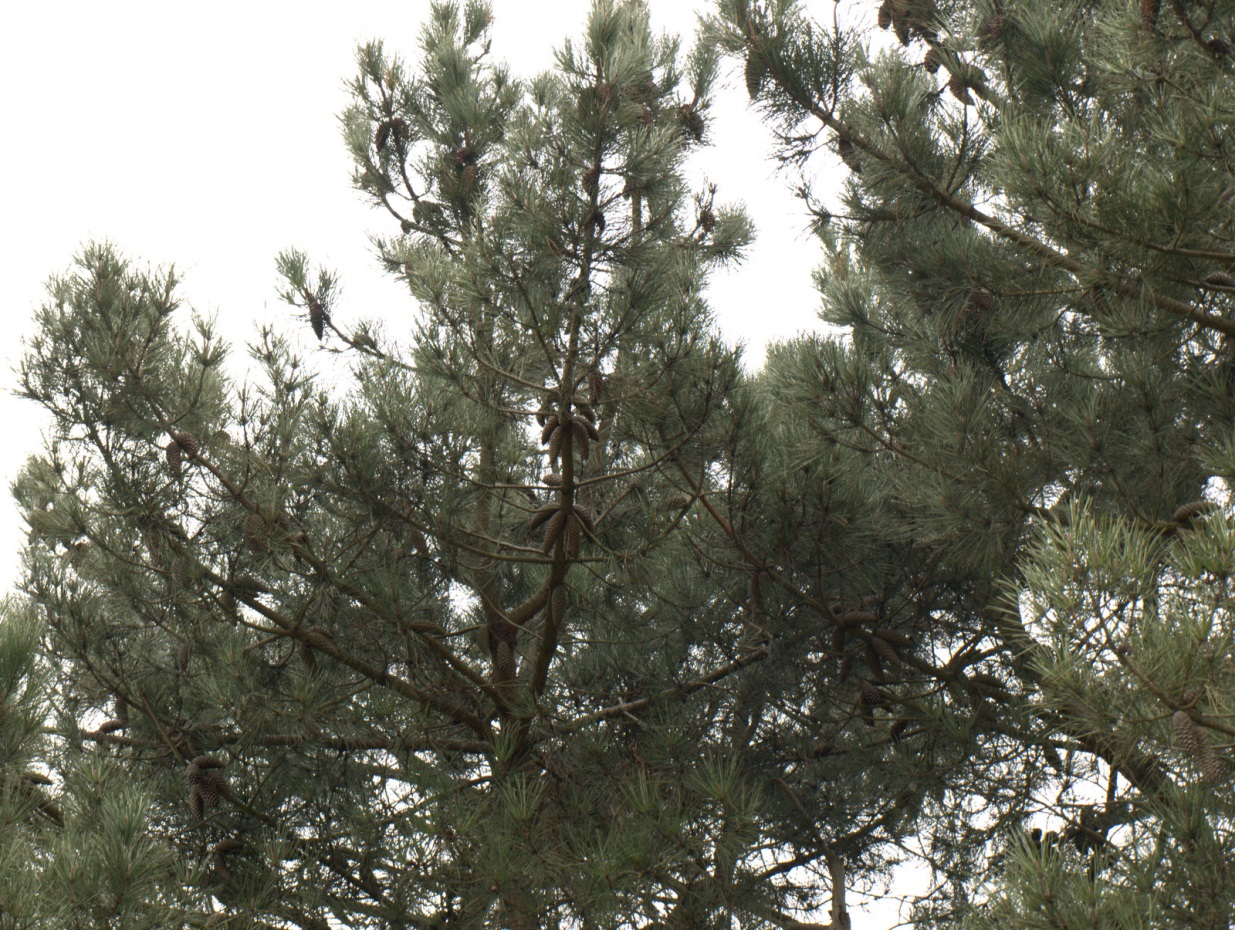


**SUPPLEMENTARY TABLE 1.** Posterior mode values and credible intervals (95% CI) of height (cm) per reproductive class (juvenile, vegetative, female, male or cosexual) and experimental group (selected, control) in *Pinus pinaster* trees. TSFR indicates the median threshold size for first reproduction per sex. Cells marked with an asterisk (*) indicate significant (P<0.05) differences between selected and control groups.

|  |  | selected | | |  |  | control | | | | |  |
| --- | --- | --- | --- | --- | --- | --- | --- | --- | --- | --- | --- | --- |
|  | Reproductive class | n | % | mode | 95% CI | | n | % | mode | 95% CI | | sig. |
| Height | Juvenile | 167 |  | 165.4 | 156.1 | 184.1 | 15 |  | 100.0 | 67.2 | 137.3 | * |
|  | Vegetative | 155 |  | 268.6 | 251.7 | 280.2 | 51 |  | 199.7 | 182.5 | 221.2 | * |
|  | Female | 134 | 40 | 285.1 | 264.7 | 294.1 | 42 | 33 | 237.6 | 213.4 | 254.5 | * |
|  | Male | 81 | 24 | 316.8 | 306.6 | 340.2 | 17 | 13 | 305.3 | 272.5 | 331.9 |  |
|  | Cosexual | 119 | 36 | 346.3 | 334.3 | 365.2 | 70 | 54 | 308.9 | 292.0 | 325.5 | * |
|  | Total | 656 | 51 |  |  |  | 195 | 66 |  |  |  |  |
| TSFR | Female |  |  | 322.6 | 293.0 | 362.4 |  |  | 215.7 | 187.1 | 241.7 | * |
|  | Male |  |  | 323.9 | 310.6 | 338.2 |  |  | 246.5 | 230.5 | 267.7 | * |

n, number of trees per class. Total, total number of trees per group and age. %, referred to F, M and C represents the percentage of those classes respect to reproductive trees. % referred to total represent the percentage of reproductive trees. Pines are monoecious species and individual trees usually produce both female and male cones, in our study, Female and Male trees represent protogynous and protandrous individuals. Vegetative trees where those that could reproduce according to their size, but they did not.

**SUPPLEMENTARY TABLE 2.** Genetic and phenotypic correlations between vegetative and reproductive traits measured in *Pinus pinaster* trees grown in a common garden in North Spain. Significant correlations (P< 0.05) in bold face.

**a)** Half-sib family genetic correlations (above diagonal) and corresponding P-values (below diagonal) among traits in the group selected for timber production.

|  |  | Female | |  | Male | |  | Vegetative | |
| --- | --- | --- | --- | --- | --- | --- | --- | --- | --- |
|  |  | TSFR | R-V |  | TSFR | R-V |  | Height | Stem form |
| Female |  |  |  |  |  |  |  |  |  |
|  | TSFR |  | **-0.82** |  | **0.39** | -0.19 |  | 0.12 | **-0.38** |
|  | R-V | <0.001 |  |  | **-0.52** | 0.00 |  | -0.14 | 0.25 |
|  |  |  |  |  |  |  |  |  |  |
| Male | TSFR | 0.03 | 0.00 |  |  | 0.02 |  | **0.38** | -0.31 |
|  | R-V | 0.31 | 0.99 |  | 0.90 |  |  | 0.06 | 0.02 |
| Vegetative |  |  |  |  |  |  |  |  |  |
|  | Height | 0.51 | 0.45 |  | 0.04 | 0.74 |  |  | -0.36 |
|  | Stem form | 0.04 | 0.17 |  | 0.09 | 0.92 |  | 0.05 |  |

TSFR, Threshold size for first reproduction; R-V, reproductive investment relative to size.

**b)** Phenotypic correlations among raw traits for the selected (above diagonal) and control, unselected, (below diagonal) groups. 95% credible intervals in brackets.

|  | Female | Male | H | Stem Form |
| --- | --- | --- | --- | --- |
| Female |  | 0.62 | **0.35** | 0.15 |
|  |  | (-0.06-0.85) | (0.27-0.46) | (-0.13-0.46) |
| Male | **0.76** |  | **0.52** | 0.12 |
|  | (0.53-0.92) |  | (0.44-0.58) | (-0.24-0.33) |
| Height | **0.28** | **0.45** |  | **0.52** |
|  | (0.10-0.36) | (0.45-0.66) |  | (0.31-0.54) |
| Stem Form | 0.23 | 0.12 | **0.52** |  |
|  | (-0.18-0.54) | (-0.26-0.49) | (0.44-0.58) |  |

Female, absolute female reproduction; Male, qualitative male reproduction (scale 0-3).

REFERENCES CITED IN THE SUPPLEMENTARY INFORMATION

1.

Alía, R., Martín, S., de Miguel, J., Galera, R., Agúndez, D., Gordo, J., *et al.* (1996). *Las regiones de procedencia de* Pinus pinaster *Aiton.* Ministerio de Medio Ambiente, Madrid, Spain.

2.

Hadfield, J.D. (2010). MCMC methods for multi-response generalized linear mixed models: the MCMCglmm R package. *J. Stat. Softw.*, 33, 1–22.

3.

Lamy, J.-B., Lagane, F., Plomion, C., Cochard, H. & Delzon, S. (2012). Micro-evolutionary patterns of juvenile wood density in a pine species. *Plant Ecol.*, 213, 1781–1792.

4.

Nakagawa, S. & Schielzeth, H. (2010). Repeatability for Gaussian and non-Gaussian data: a practical guide for biologists. *Biol. Rev. Camb. Philos. Soc.*, 85, 935–956.

5.

Sakai, A., Weller, S.G., Culley, T.M., Campbell, D.R., Dunbar-Wallis, A.K. & Andres, A. (2008). Sexual dimorphism and the genetic potential for evolution of sex allocation in the gynodioecious plant, Schiedea salicaria. *J. Evol. Biol.*, 21, 18–29.

6.

Varghese, M., Kamalakannan, R., Harwood, C.E.C.E., Lindgren, D. & McDonald, M.W.M.W. (2009). Changes in growth performance and fecundity of Eucalyptus camaldulensis and E. tereticornis during domestication in southern India. *Tree Genet. Genomes*, 5, 629–640.

7.

De Villemereuil, P., Gimenez, O. & Doligez, B. (2013). Comparing parent-offspring regression with frequentist and Bayesian animal models to estimate heritability in wild populations: a simulation study for Gaussian and binary traits. *Methods Ecol. Evol.*, 4, 260–275.

8.

White, T.L., Adams, W.T. & Neale, D.B. (2007). *Forest Genetics*. CABI Publishing.
